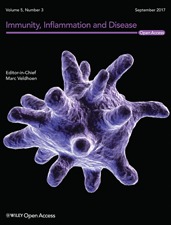# Issue Information

**DOI:** 10.1002/iid3.130

**Published:** 2017-08-24

## Abstract